# Correction: Chu et al. Inorganic Arsenic in Drinking Water and Bladder Cancer: A Meta-Analysis for Dose-Response Assessment. *Int. J. Environ. Res. Public Health* 2006, *3*(4), 316–322

**DOI:** 10.3390/ijerph15050929

**Published:** 2018-05-07

**Authors:** Huei-An Chu, Douglas Crawford-Brown

**Affiliations:** 1 State of California Energy Commission, Sacramento, CA 95814-5512, USA; ann.chu@energy.ca.gov; 2 Department of Land Economy, University of Cambridge, Cambridge CB3 9EP, UK; 3 Department of Environmental Sciences and Engineering, University of North Carolina at Chapel Hill, Chapel Hill, NC 27599, USA

In our previous paper [1], we provided details of a meta-analysis of cancer risks associated with exposure to arsenic. In an on-line addendum in 2007, we updated the results of the meta-analysis.

In the original paper, there is a brief section towards the end in which we report converting excess relative risk (ERR) to absolute risk (AR) using the results of the meta-analysis and the background rate (NR) of bladder cancer in the US. We then contrasted the resulting slope factor (SF) with that in the EPA’s IRIS database, noting that our result suggests a substantially lower value for SF. 

Unfortunately, in doing so, we used the annual incidence rate of bladder cancer, rather than the lifetime rate. This makes for an incorrect comparison against the EPA value of SF. Fortunately, colleagues at the EPA noticed this error, and so there were no (incorrect) adjustments made to the IRIS value for SF based on our analysis. However, we wish to correct the result in the original paper to avoid future mistakes, should that paper be used for further regulatory analyses.

The IRIS value for SF is 1.5 × 10^−3^ per µg/kg-day (albeit using skin cancer rather than bladder cancer). With our corrected results taking into account lifetime rather than annual incidence in the US population for mortality from bladder cancer (taken from the American Cancer Society at https://www.cancer.org/cancer/cancer-basics/lifetime-probability-of-developing-or-dying-from-cancer.html, averaged over male and female values), we obtain a value for SF of 5.5 × 10^−4^ for the mean, and 1.7 × 10^−3^ for the upper 95th percentile of the meta-analysis. Note that this interval contains the EPA’s IRIS value. The same correction applies to Figures 5 and 6 and Table 3 on page 321. Our values for SF increase by a factor of approximately 4 if one considers morbidity as well as mortality.

We apologize for this error. It does not affect the results of the meta-analysis, which was the main feature of the paper, but it has significant implications for regulatory risk assessment of arsenic exposures, and for any regulation of arsenic in ingested environmental media. We thank colleagues for calling this error to our attention. 
